# Generation of a pdmH1N1 2018 Influenza A Reporter Virus Carrying a mCherry Fluorescent Protein in the PA Segment

**DOI:** 10.3389/fcimb.2021.827790

**Published:** 2022-01-20

**Authors:** Ling Bu, Boqian Chen, Lei Xing, Xuejun Cai, Shuhua Liang, Liying Zhang, Xinhua Wang, Wenjun Song

**Affiliations:** ^1^ State Key Laboratory of Respiratory Disease, National Clinical Research Center for Respiratory Disease, Guangzhou Institute of Respiratory Health, The First Affiliated Hospital of Guangzhou Medical University, Guangzhou, China; ^2^ Institute of Integration of Traditional and Western Medicine, The First Affiliated Hospital of Guangzhou Medical University, Guangzhou, China; ^3^ Artemisinin Research Center, Guangzhou University of Chinese Medicine, Guangzhou, China; ^4^ KingMed School of Laboratory Medicine, Guangzhou Medical University, Guangzhou, China; ^5^ State Key Laboratory for Emerging Infectious Diseases, Department of Microbiology, and the Research Center of Infection and Immunology, The University of Hong Kong, Hong Kong, Hong Kong SAR, China

**Keywords:** influenza A virus, fluorescent virus, reporter virus, H1N1, mouse adaptation

## Abstract

Influenza A virus (IAV) is a major human pathogen associated with significant morbidity and mortality worldwide. Through serial passage in mice, we generated a recombinant pdmH1N1 2009 IAV, A/Guangdong/GLW/2018 (GLW/18-MA), which encodes an mCherry gene fused to the C-terminal of a polymerase acidic (PA) segment and demonstrated comparable growth kinetics to the wild-type. Nine mutations were identified in the GLW/18-MA genome: PA (I61M, E351G, and G631S), NP (E292G), HA1 (T164I), HA2 (N117S and P160S), NA (W61R), and NEP (K44R). The recombinant IAV reporter expresses mCherry, a red fluorescent protein, at a high level and maintains its genetic integrity after five generations of serial passages in Madin-Darby Canine Kidney cells (MDCK) cells. Moreover, the imaging is noninvasive and permits the monitoring of infection in living mice. Treatment with oseltamivir or baicalin followed by infection with the reporter IAV led to a decrease in fluorescent protein signal in living mice. This result demonstrates that the IAV reporter virus is a powerful tool to study viral pathogenicity and transmission and to develop and evaluate novel anti-viral drugs, inhibitors, and vaccines in the future.

## Introduction

Influenza A virus (IAV) is the most widespread virus worldwide, which causes respiratory diseases in avian and mammalian host species (Webster, 2002). To date, pandemic H1N1 (pdmH1N1/2009) and H3N2 subtypes co-circulate with type B viruses worldwide (Phipps et al., 2017). However, outbreaks of avian influenza viruses (i.e., H5N1, H7N9, and H9N2) in humans, although not resulting in a pandemic, show that exposure to infected poultry is the main risk factor for infection (Centers for Disease, C. and Prevention, 1997; Song and Qin, 2020; Song et al., 2021). Moreover, the IAV-segmented genome structure easily undergoes reassortment among hosts and among various subtypes, which may result in novel pandemic strains that threaten human health (Steel and Lowen, 2014).

The latest development of *in vivo* bioluminescence or fluorescence imaging technology enables scientists to directly monitor cell activity in living organisms (Kim et al., 2015). Bioluminescence uses the luciferase gene to label cells or DNA, while fluorescence technology uses fluorescent reporter groups (i.e., eGFP, RFP, mCherry, or Venus) without the need to inject substrates for imaging (Perez et al., 2013). The use of sensitive optical detection equipment allows researchers to directly analyze molecules, cells, and tissues from a range of living systems and investigate their real-time dynamics.

One of the useful approaches used to monitor how the virus replicates in cells in real-time is to generate reporter-expressing influenza viruses which are engineered to express bioluminescent or fluorescent proteins in the polymerase genes, surface genes, or non-structural genes (Pan et al., 2013). However, most of the reporter viruses generated are derived from laboratory strains, such as A/Puerto Rico/8/1934 or A/WSN/33, which are atypical since they lack glycosylation sites on the HA segments, and are hard to reflect pathogenicity to contemporary humans (DiPiazza et al., 2017). Recently, a mouse-adapted pdm09 H1N1 (A/CA/4/2009) was used to generate a reporter virus that expressed a NanoLuc luciferase gene (Cai et al., 2018). However, the description of the generation of this reporter virus was unclear.

An IAV is encapsulated by a lipid bilayer envelope, and the viral particle is composed of eight single-stranded RNA segments (vRNA) (Webster et al., 1992). Each vRNA segment is separately encapsulated by multiple copies of NP protein forming a twisted panhandle structure. Three polymerase proteins (PB2, PB1, and PA) bind to the non-coding regions (NCRs) or untranslated regions (UTRs) to form an RNP complex. Each segment contains a coding region, which encodes one or two viral proteins, flanked by 5’- and 3’-terminal UTRs (Noda and Kawaoka, 2010). A selective packaging hypothesis emphasizes the importance of each of the eight segments for viral propagation. The packaging region of each viral segment includes 5’- and 3’-terminal UTRs and partial coding regions (Gerber et al., 2014; Seshimo et al., 2021). It has been reported that the influenza A virus can insert foreign genes into polymerase, surface, or nonstructural segments to express artificial proteins with virus replication but decrease virus replication efficiency (Pan et al., 2013; Tran et al., 2013; Fukuyama et al., 2015; Chiem et al., 2019). However, gene manipulation to a human IAV segment may fail to produce a recombinant virus unless the integrity of the packaging signal region is retained (Li et al., 2021).

A previous study used a mouse model to elucidate adapted mutations related to host interactions and viral pathogenicity through experiments in which mice were infected with human IAVs (Brown et al., 2001). However, seasonal pdmH1N1 and H3N2 human isolates replicate poorly and show no symptoms in mice (Ilyushina et al., 2010; Baz et al., 2019). Therefore, the serial lung-to-lung passage is necessary for selecting mouse-adapted human IAVs, which results in mutations in multiple genes (Ilyushina et al., 2010; Boivin et al., 2012; Choi et al., 2020). HA segment substitutions, a major factor in determining viral virulence or infectivity, are common; these affect the preference of host receptor binding (Keleta et al., 2008) or are associated with the loss of glycosylation sites (Chen et al., 2007). Furthermore, substitutions in polymerase genes have been commonly identified in mouse-adapted strains and are related to enhanced virulence in mice (Ping et al., 2010; Zhang et al., 2017). These mutations are considered crucial markers of mammalian virulence and adaptation, possibly through changing the stereo structure to affect polymerase activities in mice.

Several anti-influenza drugs that have good activities against a broad spectrum of influenza viruses, including neuraminidase inhibitors, M2 ion channel inhibitors, and polymerase inhibitors, have been licensed for the treatment of seasonal flu and pandemic infections (Pielak et al., 2009; McKimm-Breschkin, 2013; Jones et al., 2020). Tamiflu™ (the phosphate salt of oseltamivir) developed by Gilead Sciences, Inc. and F. Hoffman–La Roche Ltd is a popular orally available anti-flu drug, which is well absorbed and rapidly cleaved by endogenous esterase *in vivo*. However, the appearance of flu variants with acquired drug resistance led to the development of novel anti-flu inhibitors or chemical components for preventing future pandemics (Wu et al., 2017). Baicalin is a type of flavonoid extracted from the dried roots of the traditional Chinese herbal medicine *Scutellaria baicalensis* and possesses potent inhibitory activity against the neuraminidase of IAV (Chu et al., 2015).

In this study, we used a clinically isolated pdmH1N1 2009 influenza virus – A/Guangdong/GLW/2018 (GLW/18) – to generate a mouse-adapted strain followed by insertion of an external red mCherry gene to produce a fluorescent influenza reporter virus, GLW18-MA-mCherry. Two known chemical compounds, oseltamivir and baicalin, were applied to evaluate the inhibitory effects on the reporter virus. This reporter virus is suitable for *in vivo* imaging assay by examining its fluorescence intensity, which can be extended to carry out viral pathogenicity and transmission studies and screen anti-viral drugs using a mouse model.

## Results

### Adaptation of a pdmH1N1 2018 Variant in Mice

**Figure 1:**
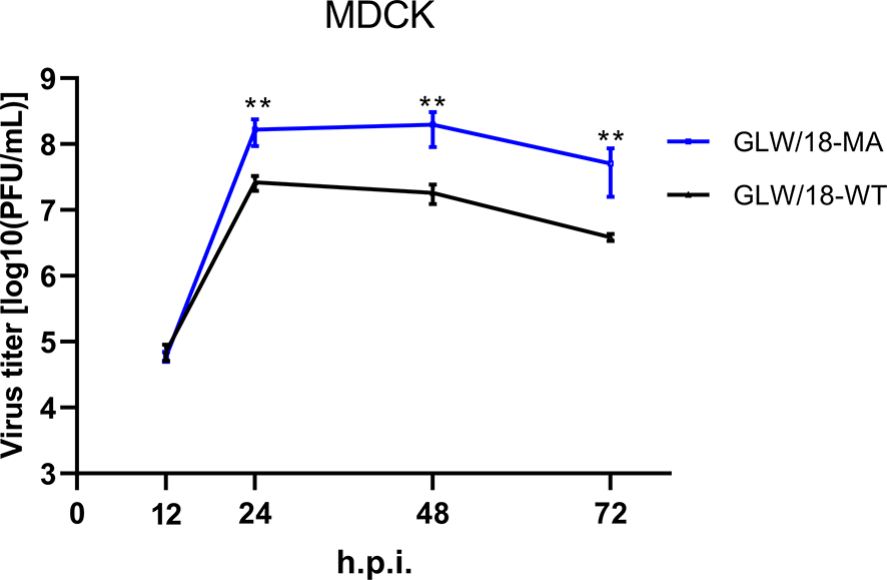
Growth curves of the wild-type and mouse-adapted GLW/18 recombinant viruses. The growth kinetics of the wild-type GLW/18 RG virus and the mouse-adapted viruses in MDCK cells were compared. The cell culture supernatants of MDCK cells infected at a multiplicity of infection (MOI) of 0.001 were collected at 12, 24, 48, and 72 h post-infection and subjected to plaque assay in MDCK cells to determine virus titers. The values displayed are the log_10_ means ± s.d. from three separate experiments. Statistical significance was determined by Student’s t-test. **P < 0.01.

Mice are almost avirulent to original pandemic H1N1 (pdmH1N1) isolates (Ilyushina et al., 2010). Therefore, to increase its virulence in mice, we generated a mouse-adapted mutant strain of the A/Guangdong/GLW/2018 (H1N1, GLW/18) by serial lung-to-lung blind passages, initialized by intranasal inoculation of 1.0 × 10^5^ PFU in 25 µL of phosphate buffered saline (PBS). After 12 passages, mice demonstrated a trend toward weight loss, suggesting that GLW/18 acquires adaptative mutations, which allows it to replicate effectively *in vivo*. We then passaged for a further three rounds. The viral RNA of the 15th generation isolate extracted from the lung homogenate was reverse-transcribed to generate cDNA, whose genome sequences were determined and named GLW/18-MA. GLW/18-MA exhibited greater virulence compared to the wild-type virus. Growth kinetics data demonstrated that the viral population of the GLW/18-MA grew approximately 10 times higher than the wild-type in 24-, 48-, and 72-hours post-infection, respectively, indicating that the mouse-adapted variant acquired substitutions in mice (**Figure 1**). Genome sequence comparison showed that nine mutations were identified in the mouse-adapted genome (GLW/18-MA). Four substitutions were related to virus replication (PA I61M, PA E351G, PA G631S, and NP E292G); another four were located at the surface gene (HA1 T164I, HA2 N117S, HA2 P160S, and NA W61R); and one was relevant to nuclear export (NEP K44R) (**Table 1**). It was reported that the mutation I to T at position PA-61 affected PA endonuclease activity to cleave host capped pre-mRNAs (Hu et al., 2016). In this study, the substitution I to M at position PA-61 might have a similar function in mice. The substitution T to I at position HA1-164 disappears a glycosylation site around the epitope Ca, which might result in the conformational change of the epitope. Hu et al. reported that HA2-117 is a key amino acid residue that affects HA stability (Hu et al., 2020). The mutation N to S at position HA2-117 in the GLW/18-MA might also increase the stability of the HA trimer for better replication and transmission in mice. These results indicated that the GLW/18-MA isolate had acquired mutations that significantly affected virulence in mice.

**Table 1:** Amino acid substitutions identified in mouse-adapted GLW/18 pdmH1N1 isolate.

Gene	Residue at position no.	Wild-type	Substitution
PA	61	I	M
	351	E	K
	631	G	S
HA1	164	T	I
HA2	117	N	S
	160	P	S
NP	292	E	G
NA	61	W	R
NEP	44	K	R

### Distribution of Mouse-Adapted Mutations in Natural Isolates

To examine whether the amino acid mutations identified in GLW/18-MA have the potential to occur in nature, we compared its genome sequence with that stored in the Global Initiative on Sharing Avian Influenza Data (GISAID). In total, we downloaded 61,737 human pdm09 strains from April 2009 to November 2021. The mouse-adapted substitutions were found in nature; I61M was found in the PA protein of 1.7% of contemporary isolates, and one glycosylation site of HA1 T164 was mutated, which occupied approximately 0.1% in the database. Both PA I61M and HA1 T164I substitutions appeared after 2017, indicating that such mutations might be co-evolved with other amino acids. The other seven mouse-adapted mutations were rare in the GISAID database.

### PA Substitutions Increased Virus Replication

**Figure 2:**
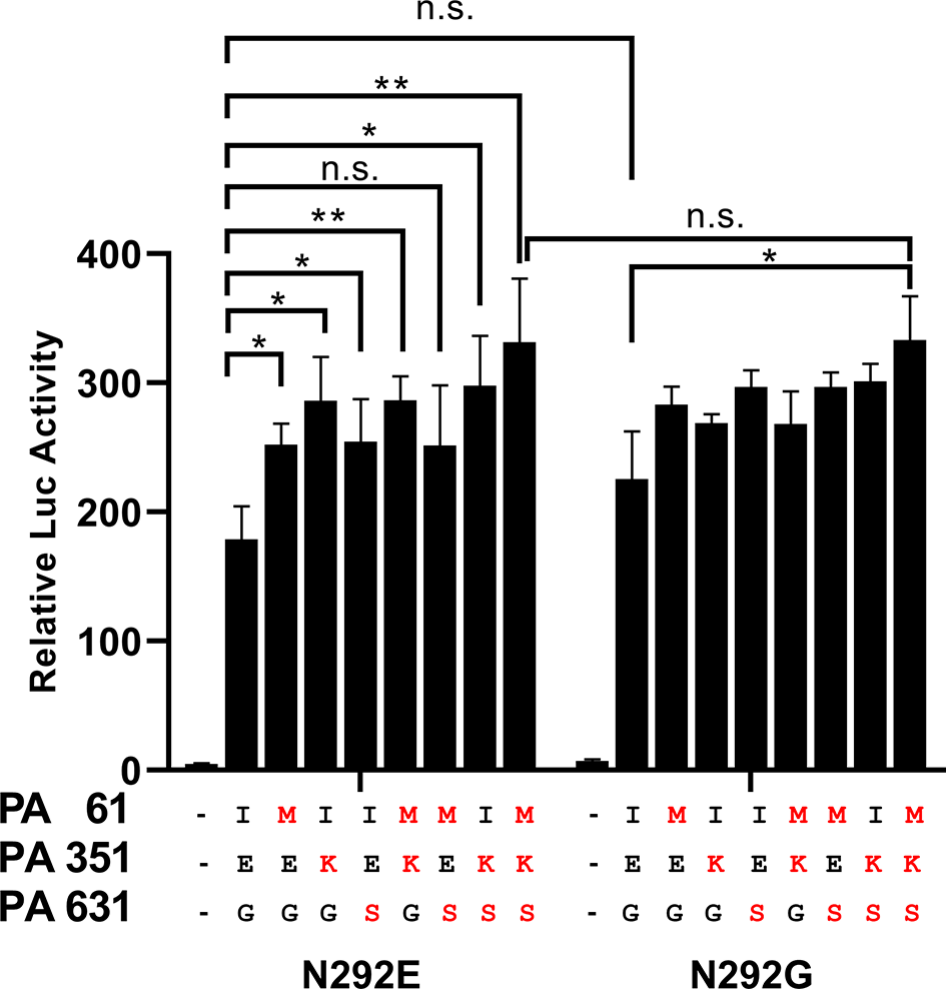
Polymerase activity of GLW/18 variants *in vitro*. HEK293T cells were transfected with GLW/18 complexes composed of wild-type genotypes of pHW2000 derived plasmids PB2, PB1, PA, and NP, and pHW2000 substitutive PA plasmids, as shown in , with the firefly luciferase reporter pYH-Luci and a Renilla luciferase reporter (internal control). Luciferase activity was measured at 24 h post-transfection, following incubation at 33°C. Statistical significance was analyzed by one-way analysis of variance, corrected by the Bonferroni post-test. **P < 0.01, *P < 0.05. n.s., no significant.

We tested whether the presence of substitutions altered the ribonucleoprotein (RNP) complex polymerase activity of the GLW/18 strain in human embryonic kidney 293T (HEK293T) cells. It was found that three PA substitutions (I61M, E351K, and G631S), either individually or together with other mutations, demonstrated higher polymerase activity than wild-type. The wild-type PA and NP E292G RNP complex showed higher polymerase activity than that of the wild-type group, but no significant difference (P value = 0.0501). Although the single mutation of PA with NP E292G did not increase the polymerase activity significantly, the PA triple substitutions with NP E292G contributed to the polymerase activity. Overall, three PA substitutions, individually or combinatorically, but not NP E292G, increased the polymerase activities of the GLW/18-MA which might affect its virulence *in vivo* (**Figure 2**).

### Effect of HA Substitutions on Antigenic Specificity

To assess whether the amino acid changes identified in the GLW/18-MA affected its antigenic specificity due to acquired HA mutations, an HI assay was performed to test the antigenic properties of wild-type and GLW/18-MA using antiserum collected from GLW/18-infected patients (Xing et al., 2021). The HI titer of the wild-type was 2560, while that of the variant was only 40, suggesting that the reactivity of the antiserum against GLW/18-MA was dramatically decreased (**Table 2**). Of the three substitutions, HA1-164 was an N-glycosylation site (NQT) in the wild-type isolate, which is located at the antigenic site Ca (Kao et al., 2012). The loss of the N-glycosylation site might change the epitope of HA and increase the fitness of the variant in mice.

**Table 2:** Antigenic characterization of GLW/18 wild-type and mouse-adapted variant by hemagglutination inhibition assay.

Residue (H3 numbering)	HA1	HA2		GLW/18 antisera against recombinant virus
	164	117	160	
GLW/18	T	N	P	2560
GLW/18-MA	I	S	S	40
GLW/18-MA-mCherry	I	S	S	40

### Effect of NA Substitution

To characterize whether NA substitution contributed to increasing the virulence of the GLW/18-MA in mice, a NA activity assay was performed using a neuraminidase assay (Beyotime) according to the manufacturer’s instruction. Three dosages (1, 0.5, and 0.25 TCID50) were applied in the assay, but it did not demonstrate the significant difference between the two groups, indicating that NA W61R substitution did not affect the virulence of the mouse-adapted variant (**Supplementary Figure 1**).

### Generation of a GLW/18-MA-mCherry Reporter Virus

**Figure 3:**
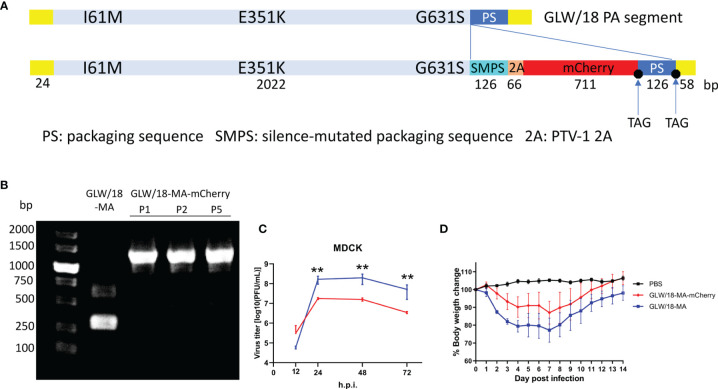
*In vitro* and *in vivo* growth characteristics of the reporter virus. **(A)** Diagram of the GLW/18 mouse-adapted PA segment engineered to express mCherry fluorescent protein during infection. **(B)** Detection of the transgenic segment in the GLW/18-MA-mCherry reporter virus. Viral RNA was isolated, and RT-PCR was performed to amplify the C-terminal of wild-type and transgenic PA segments. Forward primer: 5′-TTGAGAGCATGATTGAAGCC-3′; reverse primer: 5′-AGTAGAAACAAGGTACTTTTTTGGAC-3′. Following viral growth at 33°C for 3 days, allantoic fluid was isolated, diluted 10^−6^, and injected back into three eggs; this was repeated for a total of five passages. **(C)** The growth kinetics of the reporter virus and the mouse-adapted RG virus in MDCK cells were compared. The cell culture supernatants of MDCK cells infected at an MOI of 0.001 were collected at 12, 24, 48, and 72 h post-infection and subjected to plaque assay in MDCK cells to determine virus titers. The values displayed are the log_10_ means ± s.d. from three separate experiments. Statistical significance was determined by Student’s t-test. **P < 0.01. **(D)** To test virus replication in mice, groups of five BALB/c mice were each inoculated intranasally with 1 × 10^5^ PFU in 25 µL phosphate buffered saline (PBS). Mice were observed daily for changes in body weight for 14 days.

It has been reported that the insertion of external genes at the C-terminal of polymerase genes does not affect viral propagation unless the packaging signals or functions of the native viral polymerase are disrupted (Marsh et al., 2008; Gao et al., 2010; Hamilton et al., 2018). Briefly, influenza codon-optimized mCherry nucleotide sequences were synthesized and fused before the packaging sequence of the PA segment (Puigbo et al., 2007). A PTV-1 2A autoproteolytic cleavage site peptide triggered by ribosomal skipping of the peptide bond was inserted between the PA and mCherry coding sequences to translate two separate peptides (**Figure 3A**). Synonymous changes were introduced to inactivate the PA ORF region packaging signals. The packaging region, including the PA native packaging sequences, the stop codon, and the 5’ non-coding region (5’ NCR), retained its integrity.

We adopted Dr. Peter’s approach to generate a reporter influenza A virus (Gao et al., 2010). To this end, we made 13 PA constructs whose packaging signals on the coding region ranged from 108 nt to 138 nt (excluding stop codons). Two plasmids whose packaging sequences contained 126 and 129 nt, respectively, were successfully rescued using recombinant reporter viruses in an eight-plasmid influenza A virus rescue system (see *Materials and Methods*) (Hoffmann and Webster, 2000). The reporter virus carrying 126 nt of the PA packaging sequence was named GLW/18-MA-mCherry. Interestingly, the packaging region of GLW/18-MA was slightly different from that of PR8. The latter included 123 nt (not including stop codons) in the PA ORF region (Gao et al., 2010), while the former (including GLW/18-MA-mCherry) included 126 nt packaging sequences in the PA coding region, respectively, showing that the packaging signals are crucial for the viability of the virus.

### Characterization of Recombinant Reporter Virus

To test the genetic integrity and stability of the reporter virus, the reporter virus was continuously passaged for five generations *in ovo*. Briefly, following viral growth in 8- to 10-day-old SPF eggs at 33°C for 3–4 days, allantoic fluid was isolated, diluted 10^−5^ to 10^−6^, and used to inoculate another egg. Genome sequencing revealed that no mutations were present in the PA-mCherry segment, indicating that the insertion appears stable for at least five passages *in ovo* (**Figure 3B**).

To examine the phenotype of mouse-adapted reporter virus, a comparison of the growth kinetics of three viruses revealed that the GLW/18-MA-mCherry reporter virus replicated at a titer approximately one log lower than the mouse-adapted strain (GLW/18-MA) in MDCK cells, but grew similar to the GLW/18 wild-type (**Figure 3C**).

To further characterize the pathogenicity of the reporter virus, both GLW/18-MA and GLW/18-MA-mCherry viruses were tested in a mouse infection model. We calculated the 50% mouse lethal dose (MLD50) of both viruses; the GLW/18-MA was 10^5.25^ PFU and the GLW/18-MA-mCherry was 10^5.5^ PFU. Mice were infected with a sub-lethal dose (10^5.0^ PFU in 25 µL PBS) and changes in body weight were recorded over the following 14 days. Viruses carrying mCherry reporter genes caused moderated body weight loss, while GLW/18-MA caused more serious body weight loss, which peaked at around 20% on day 7 post-infection (**Figure 3D**).

### Calculation of TCID50 and EC50

To test whether the fluorescent virus can be used as a reporter system *in vitro and in vivo*, we selected two known anti-influenza NA inhibitors, oseltamivir (Roche) and baicalin (Chengdu Aifa, purity > 98%), in this study.

To determine the virus infectivity to MDCK cells, we calculated the median tissue culture infective dose (TCID50) using the CPE observation and HA assay methods, whose values were 10^-6.25^/100 µL for the GLW/18-MA-mCherry reporter virus and 10^-5.25^/100 µL for the GLW/18-MA virus (**Supplementary Figures 2A, B**). To define the half-maximal concentration of a drug, the half-maximal effective concentration (EC50) of oseltamivir and baicalin to MDCK cells was calculated as 1.293 and 36.64 µg/mL, respectively, using the standard method (**Supplementary Figures 3A, B**).

### Western Blotting and Immunofluorescent Assay

**Figure 4:**
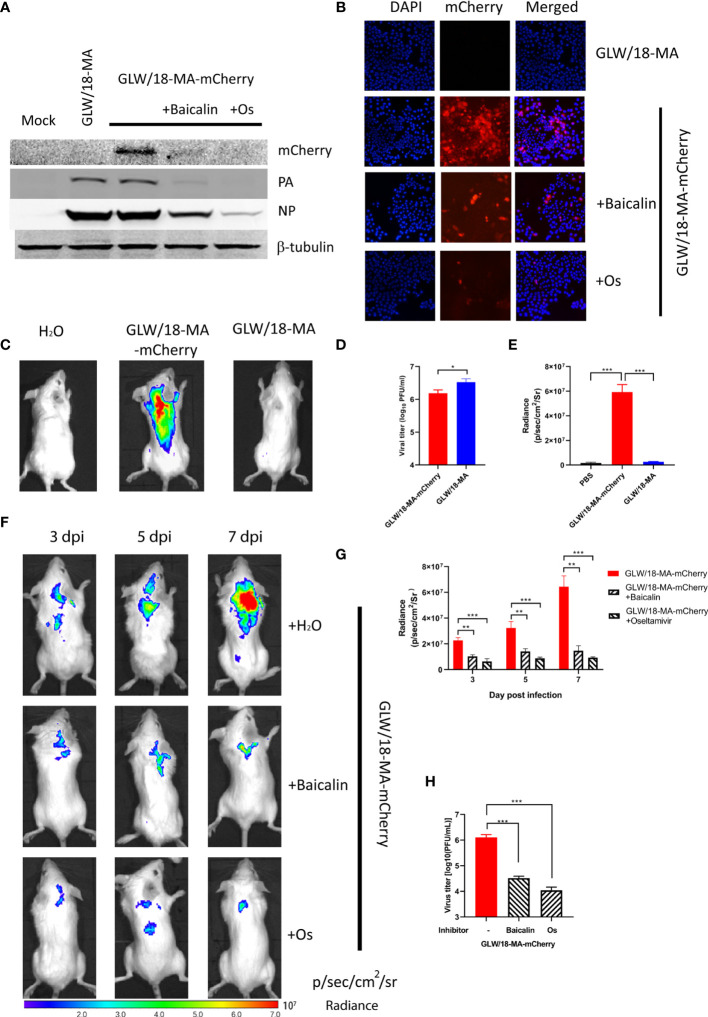
Inhibitory effect of baicalin and oseltamivir against the reporter virus. **(A)** Confluent MDCK cells were absorbed with 100 TCID50 GLW/18-wild-type or reporter virus for 2 h followed by treatment with 3.15 µM oseltamivir or 30.99 µM baicalin, respectively. Viruses and chemical compounds were cultured at 33°C for 24 h before cell lysis for western blotting detection. **(B)** MDCK cells were infected with wild-type or mCherry-expressing reporter virus at an MOI of 1. Infected cells were processed for microscopy at 8 h post-infection. After fixation, imaging of cells was performed using a fluorescent microscope (Nikon Ti-S). DAPI (4’,6’-diamidino-2-phenylindole) was used to visualize the nuclei of the cells. **(C)** Mice were infected with 1 × 10^5^ PFU viruses and analyzed for fluorescent signals at 7 d. p. i. **(D)** viral titers in lung homogenates were determined by plaque assay. **(E)** fluorescent signals of mice described for **(C)** were quantified (n=3). **(F)** To further examine the inhibitory efficiency *in vivo*, three groups of mice were intragastrically treated with oseltamivir phosphate (50 mg/kg), baicalin (150 mg/kg), or sterile water 2 days before infection. After infecting an inoculum of 1 × 10^5^ PFU reporter viruses, inhibitors were daily treated until 7 d. p. i. (n=3), images were captured at 3, 5, and 7 d. p. i., respectively. **(G)** quantification of fluorescent signals of three groups of mice infected with reporter virus untreated or treated inhibitors. **(H)** Three groups of mice in panel F were sacrificed at 7 d. p. i. for calculation of the number of viral particles in lung homogenates. ***P < 0.001, **P < 0.01, *P < 0.05.

To examine the inhibitory effect, confluent MDCK cells were infected with reporter virus using a 100TCID50 dosage. After absorption for 2 h, 3.15 µM of oseltamivir or 82.09 µM of baicalin were added and incubated at 33°C for 24 h. Cell lysis was examined by western blotting and immunofluorescent assay (IFA). It was shown that both oseltamivir and baicalin efficiently inhibited viral propagation (**Figures 4A, B**).

### *In Vivo* Imaging of Reporter Virus Infection

Each group of eight Balb/c mice was intranasally infected with an inoculum of 1 × 10^5^ PFU of the reporter virus, GLW/18-MA-mCherry. The body weight of the infected groups began decreasing at 2 days post-infection (data now shown). The fluorescent output reached a maximum at 7 days post-infection (**Figure 4C**). No obvious fluorescent signals were detected in the groups of mock infection and GLW/18-MA. To examine the virus replication ability in mice lung tissue, three mice from two groups were euthanized on day 7 post-infection and lung tissue was collected for virus titration. Consistent with the body weight loss and growth kinetic results, mouse-adapted viruses replicated twice as fast as the mCherry reporter virus *in vivo* (**Figure 4D**). Both groups infected with mouse-adapted viruses had clear lesions, but mice infected with mCherry-expressed reporter viruses were visualized robust fluorescence (**Figure 4E**). *In vivo* imaging data demonstrated that mCherry-expressed influenza A virus could be used to monitor the animal infection.

To further examine the inhibitory efficiency *in vivo*, three groups of mice were intragastrically treated with oseltamivir phosphate (50 mg/kg), baicalin (150 mg/kg), or PBS 2 days before infection. After infecting an inoculum of 1 × 10^5^ PFU reporter viruses, inhibitors were given daily until 7 days post-infection. As expected, mice infected with GLW/18-MA-mCherry demonstrated increased fluorescent signals over time. In total, images were captured at 3, 5, and 7 d. p. i., respectively. However, the fluorescent outputs of the inhibitor-treated groups were much weaker than the untreated group (**Figure 4F**). The fluorescent signals of three groups were quantified (**Figure 4G**). The number of viral particles from lung homogenates was consistent with the fluorescent signals at 7 days post-infection (**Figure 4H**).

*In vivo* imaging data demonstrated that the mCherry-expressed influenza reporter virus could monitor animal infection. Thus, this method provides an approach for the study of viral pathogenicity and the development and evaluation of new anti-viral drugs, inhibitors, and vaccines in the future.

## Materials And Methods

### Viral Propagation

Viruses were propagated in 8-to 10-day-old SPF eggs or MDCK cells for 3-4days at 33°C. Viral RNAs (vRNAs) were extracted to prepare single-strand complementary DNA (ss cDNA) for amplification of viral segments (QIAGEN).

### Construction of Recombinant Reporter Viruses

All of the influenza A viruses applied in this study were rescued using reverse genetics (RG) techniques to ensure the absence of contamination from other microbes (Hoffmann and Webster, 2000). The viral plasmids were constructed as described previously (Xing et al., 2021). The mCherry gene was cloned into pHW2000-GLW-PA plasmid. To avoid redundancy of packaging sequences within the PA vRNA segment, silent mutation of packaging sequences (SMPS) within the terminal 126 nt of PA was applied, and the original PA packaging sequences were fused downstream of the mCherry gene. A 2A peptide from porcine teschovirus 1 (PTV-1 2A) was inserted between PA and mCherry for the translation of two independent proteins.

### Minigenome Reporter Assays

Luciferase activity-based minigenome reporter assays were performed as described previously (Huang et al., 2017). RNP complexes composed of PA, PB1, PB2, and NP derived from A/Guangdong/GLW/2018-wild-type and mouse-adapted strains and cloned into the pHW2000 vector (50 ng each) were mixed with a luciferase reporter plasmid (50 ng) and a thymidine kinase promoter-Renilla luciferase reporter plasmid (pRL_TK) construct (10 ng). Subsequently, these constructs were co-transfected into HEK293T cells and incubated at 33°C, supplied with CO_2_. The luciferase activity was measured using a Dual-Luciferase Reporter Assay System (Promega) at 24 h post-transfection. The RNP polymerase activity was normalized against pRL_TK activity.

### Viral Growth Kinetics in Cells

Confluent MDCK cells were infected with RG viruses at a multiplicity of infection (MOI) of 0.001. The viral inocula were removed after 1 h of adsorption at 33°C and replaced with minimal essential medium (MEM) containing 1 mg/mL of L-(tosylamido-2-phenyl) ethyl chloromethyl ketone (TPCK)-treated trypsin. Infected cells were further incubated at 33°C, supplied with 5% CO_2_. Culture supernatants were collected at different time points post-infection and viral titers were determined by the plaque assay in MDCK cells. Culture supernatants were collected at different time points post-infection and viral titers were determined by the standard 50% tissue culture infective dose assay in MDCK cells.

### TCID50 Determination of Viruses

MDCK cells were grown in 96-well plates for 24 h. The virus stock was serially diluted 2-fold by serum-free media. Then, 100 µL of serial virus dilution was added to each well at 33°C for 2 h. The cells were incubated in a maintenance medium at 33°C for another 48 h. The cell viability was measured by hemagglutination assay. The TCID50 (50% tissue culture infectious dose) was determined using the Reed-Muench method (Reed and Muench, 1938).

### HI Assay

HI tests with recombinant viruses were conducted as described previously (Wu et al., 2008). Briefly, recovered patient sera collected from the GLW/18-infected patient were treated with receptor-destroying enzyme (RDE, Denka Seiken Co., Tokyo, Japan). Control antibodies and sera were then 2-fold serially diluted in 96-well plates. Subsequently, 8 HA units of the virus were added to each well and incubated at room temperature for 1 h. Then, 50 μL of 0.5% Turkey erythrocytes (Lampire Biological Laboratories, PA) was added to the serum/virus mixture followed by incubation for 30 min. The HI titer is defined as the reciprocal of the highest dilution of sera that completely inhibits hemagglutination. The HI test was started at a 1:20 dilution for anti-GLW/18 serum.

### NA Activity Assay

NA activity was detected by a Neuraminidase Assay Kit (Beyotime), following the manufacturer’s instructions. Briefly, the same TCID50 doses of wild-type or reporter viruses were added to 70 μL detection buffer, followed by adding 10 μL water and 10 μL NA fluorescent substrate. After incubation at 37°C for 30 min, the fluorescence intensity was measured at an excitation of 322 nm and an emission of 450 nm using a Synergy X Multi-Mode Microplate Reader (Bio-Tek, Winooski, Vermont, USA).

### Preparation of Chemical Compounds

Baicalin and oseltamivir were dissolved in 60°C sterile water and stored at 4°C.

### Replication in Mice and Lethal Dose Determination

For replication in mice and lethal dose determination, female BALB/c mice, aged 5–6 weeks, were obtained from Beijing Huafukang Biotechnology Co., Ltd. For MLD50 determination, groups of five mice were anesthetized with isoflurane (Halocarbon Laboratory) and intranasally inoculated with 25 mL of 10-fold serial dilutions of RG virus in PBS, at a dose range of 10^4^–10^6^ PFU. Body weight and survival were monitored daily for 14 days after infection. The MLD50 of RG viruses was calculated by the Reed-Muench Method (Reed and Muench, 1938) using GraphPad Prism Version 8 software. To test virus replication in mice, groups of eight mice were anesthetized with isoflurane and inoculated intranasally with 1 × 10^5^ PFU (25 µL) of RG viruses. Animals were observed daily for mortality and body weight was measured for up to 14 days after infection. At 7 days post-infection, three mice from each group infected with wild-type or reporter virus carrying mCherry were euthanized and lung tissue samples were collected from each mouse for virus titration. *In vivo* imaging was performed using a PerkinElmer IVIS Lumina III imaging system. Imaging capture and analysis were performed by the PerkinElmer Living Imaging software. The protocols for the animal experiments were approved by the Ethics Committee of The First Affiliated Hospital of Guangzhou Medical University (File No. 2021072 approved on March 16, 2021).

### Statistical Analysis

Statistical analysis was conducted using GraphPad Prism Version 8 software (GraphPad Software Inc.).

## Discussion

Genome packaging of IAV is a fundamental process in the viral life cycle. All eight vRNAs are selectively packaged into each progeny virion through segment-specific genome-packaging signal sequences located in the noncoding and terminal coding regions of both the 5′ and the 3′ ends of the vRNAs (Goto et al., 2013). Packaging regions of the viral eight segments are proved to be crucially involved in the generation of reassorted viruses (Essere et al., 2013). Reporter viruses can be generated by inserting foreign genes into the viral segments. It has been reported that six segments of the influenza A virus, including PB2, PB1, PA, HA, NA, and NS. However, most of them were derived from the PR8 or WSN laboratory-adapted isolates, which may not be suitable for the study of contemporary epidemics (Kittel et al., 2004; Gao et al., 2010; Martinez-Sobrido et al., 2010; Pan et al., 2013; Fukuyama et al., 2015). Therefore, in this study, we selected a pdmH1N1 2018 clinical isolate to engineer its PA segment and created a fluorescent-expressed reporter virus. Interestingly, it was found that the 5’ packaging signal region of the PA segment of GLW/18 contained 187 nt (129 nt in the coding region and 58 nt in the 5’ non-coding region), which was 3 nt more than that of the PR8 strain (Gao et al., 2010). Dr. Mehle’s group generated a WSN-based PA-Nluc reporter virus, whose packaging sequences in the coding region were only 50 nt, but failed to rescue a recombinant GLW/18 reporter virus (Tran et al., 2013). Meanwhile, we engineered the PA segment of a cold-adapted H2N2 strain, A/Ann Arbor/6/1960, and identified that its packaging region was different from that of PR8, WSN, and GLW/18, respectively (data not shown). PA segments from three isolates have different packaging signals at 5’ NCR, suggesting that packaging signal regions are variable in the different subtypes. Another two basic polymerases, PB2 and PB1, also can be used a similar approach to translate a foreign mRNA like PA segment, but the insertion sizes cannot be longer than 1kbp (Heaton et al., 2013; Munier et al., 2013; Yan et al., 2015). Our unpublished data showed that, among 3 polymerase subunits, PA is the most stable gene which can express around 2k bp size of foreign gene separated by the PTV-1 2A peptide. Influenza viruses with NS1 deletions (DelNS1 influenza viruses) are another common reporter virus that expresses foreign proteins taking over the NS1 open reading frame with some modifications to prevent the splicing activity (Salvatore et al., 2002; Zheng et al., 2015). It seems that the size of the DelNS1 virus for insertion of foreign genes could not be longer than 1 kbp (Salvatore et al., 2002; Wang et al., 2019). Packaging signal regions are composed of 3’- and 5’- NCRs and partial ORFs of each viral segment. Once viral packaging signals sequences were disrupted, the reporter virus would not be successfully rescued. Therefore, the generation of reporter influenza viruses might be a good approach to study the mechanism of the selective packaging model of IAV.

In past decades, several epidemics of respiratory infections from newly emerging viruses have seriously threatened global health and the economy; such viruses include severe acute respiratory syndrome coronavirus (SARS-CoV-1 and SARS-CoV-2), Middle East respiratory syndrome coronavirus (MERS-CoV), and the pandemic influenza A (H1N1), H5N1, and H7N9 avian influenza (AI) viruses. Therefore, it is necessary to develop pandemic risk assessment tools that evaluate the pathogenicity, pandemic potential, and susceptibility to antiviral interventions of such viruses (Harrington et al., 2021). Reporter viruses expressing bioluminescent or fluorescent proteins can facilitate us to monitor virus replication in live animals noninvasively, such as PR8, WSN, CA4, and SARS-CoV-2 reporter viruses (Tran et al., 2013; Heaton et al., 2013; Cai et al., 2018; Thi Nhu Thao et al., 2020). The advantages of recombinant reporter viruses can be easily observed virus replication *in vivo* by detecting fluorescent strength expressed by inserted genes such as luciferase, eGFP or mCherry without euthanasia of rodents for detection of lung titers and acquire whole-body imaging by a non-invasion technology. However, the reporter virus usually has a lower titer and pathogenicity than its wild-type form, possibly due to the external gene that might decrease the transcriptional and translational efficiency during virus propagation. To solve this problem, the adapted strain could be passaged more generations in mice to acquire sufficient mutations for future study.

Here, we engineered a reporter influenza A virus, which replicates well and expresses a fluorescent mCherry protein to the virus *in vivo* that can be used to detect flu replication and rapidly assess the safety and efficacy of antiviral inhibitors in the same animal throughout the course of infection. Moreover, reporter viruses can be applied as a useful tool for screening of anti-viral drugs or inhibitors, identification of host factors related to viral replication, and development of live-attenuated vaccines.

## Data Availability Statement

The datasets presented in this study can be found in online repositories. The names of the repository/repositories and accession number(s) can be found below: www.gisaid.org, EPI_ISL_335963.

## Ethics Statement

The animal study was reviewed and approved by the Ethics Committee of The First Affiliated Hospital of Guangzhou Medical University (File No. 2021072 approved on March 16, 2021).

## Author Contributions

Author Contributions LB, BC, and LX performed the research and analyzed the results. XC, SL, and LZ performed virus isolation, analyzed the clinical data. WS and XW co-directed the study. BC and WS directed the study, analyzed, interpreted results, and wrote the manuscript. All authors contributed to the article and approved the submitted version.

## Funding

This work was supported by the National Natural Science Foundation of China (Grant No. 82074311, 82141211), the General Project of Guangzhou Medical University (Grant No. SKLRD-MS-201908), the Open Project of Guangzhou Medical University (Grant No. SKLRD-OP-202203), the Yunnan Provincial Science and Technology Department (Grant No. 202005AF150043), and Guangzhou Institute of Respiratory Health Open Project (Funds provided by China Evergrande Group, Project No. 2020GIRHHMS18).

## Conflict of Interest

The authors declare that the research was conducted in the absence of any commercial or financial relationships that could be construed as a potential conflict of interest.

## Publisher’s Note

All claims expressed in this article are solely those of the authors and do not necessarily represent those of their affiliated organizations, or those of the publisher, the editors and the reviewers. Any product that may be evaluated in this article, or claim that may be made by its manufacturer, is not guaranteed or endorsed by the publisher.

## Supplementary Material

Supplementary Figure 1 Neuraminidase (NA) activity assay. The NA activity of viruses was determined by a kit as described in Materials and Methods. Three dosages (1 TCID50, ½ TCID50, and ¼ TCID50) of wild-type and reporter viruses were used in the assay, and the results are displayed as means ± s.d. (n = 3).

Supplementary Figure 2 Groups of mice were infected with 25 µL inocula containing 10^5^ or 10^6^ PFU of reverse genetic versions of viruses, as specified. Mice were observed daily for changes in body weight for 14 days (day 0 to day 14). Animals that lost 25% of their pre-infection weight were euthanized, in accordance with our institutional animal ethics guidelines. The MLD50 values were calculated using the Reed and Muench method.

Supplementary Figure 3 Confluent MDCK cells cultured in 96-well plates were absorbed with 100TCID50 virus for 2 h, followed by co-culturing with baicalin (2-fold dilution from 204 μg/mL to 2.37 μg/mL) or oseltamivir (2-fold dilution from 20µg/mL to 0.156 µg/mL). The EC50 values were determined by the MTT method (Abcam) using GraphPad Prism 8.
