# Supplementary figures and images for: Generation of a pdmH1N1 2018 Influenza A Reporter Virus Carrying a mCherry Fluorescent Protein in the PA Segment
Source: Front Cell Infect Microbiol. 2022 Jan 20;11:827790. doi: 10.3389/fcimb.2021.827790 (PMC8811159; doi:10.3389/fcimb.2021.827790)

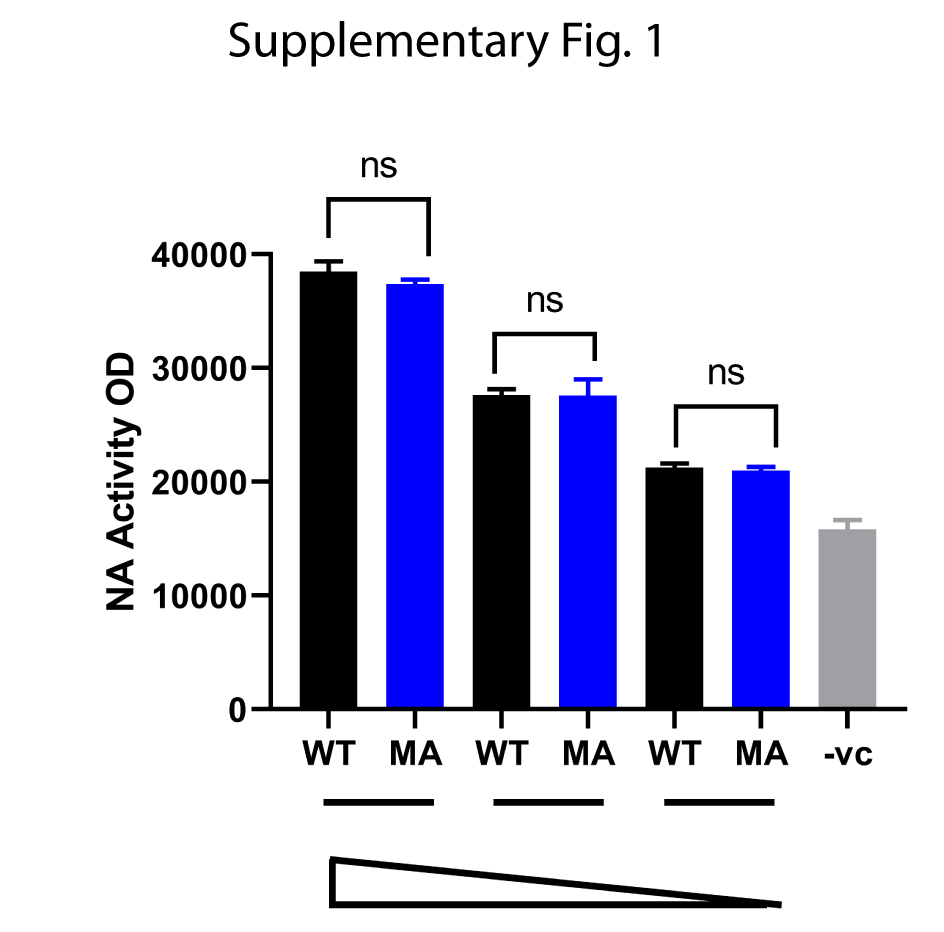

Supplement: Supplementary Figure 1 — Neuraminidase (NA) activity assay. The NA activity of viruses was determined by a kit as described in Materials and Methods. Three dosages (1 TCID50, ½ TCID50, and ¼ TCID50) of wild-type and reporter viruses were used in the assay, and the results are displayed as means ± s.d. (n = 3). [file Image_1.tif]

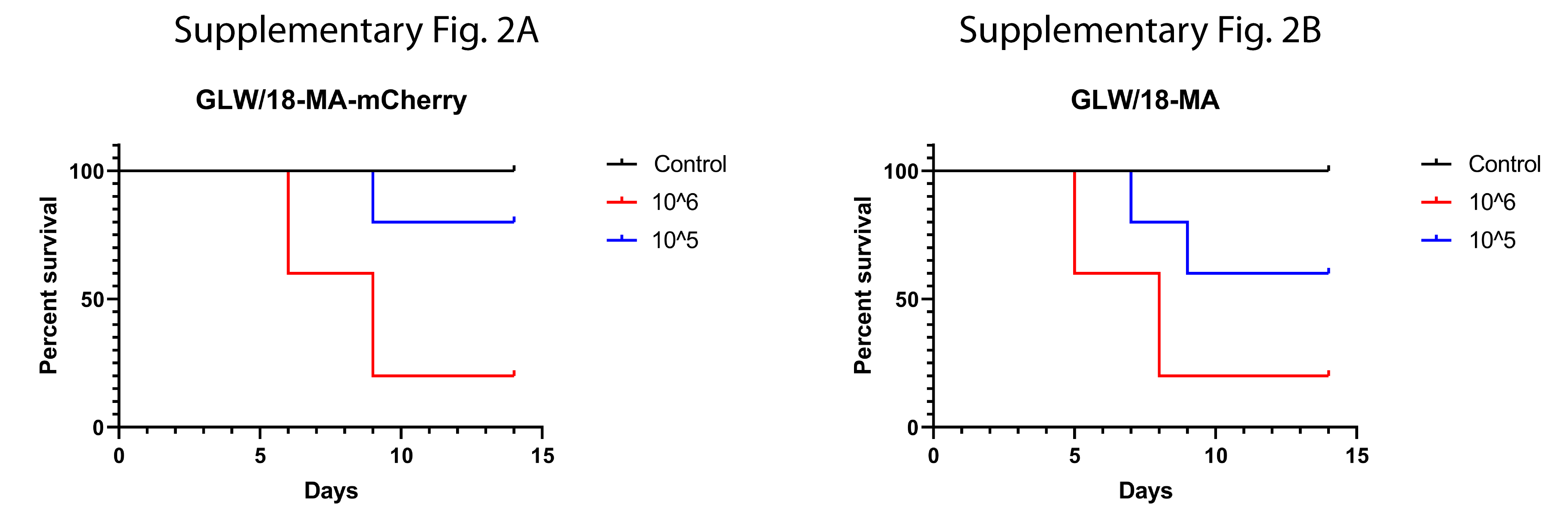

Supplement: Supplementary Figure 2 — Groups of mice were infected with 25 µL inocula containing 105 or 106 PFU of reverse genetic versions of viruses, as specified. Mice were observed daily for changes in body weight for 14 days (day 0 to day 14). Animals that lost 25% of their pre-infection weight were euthanized, in accordance with our institutional animal ethics guidelines. The MLD50 values were calculated using the Reed and Muench method. [file Image_2.tif]

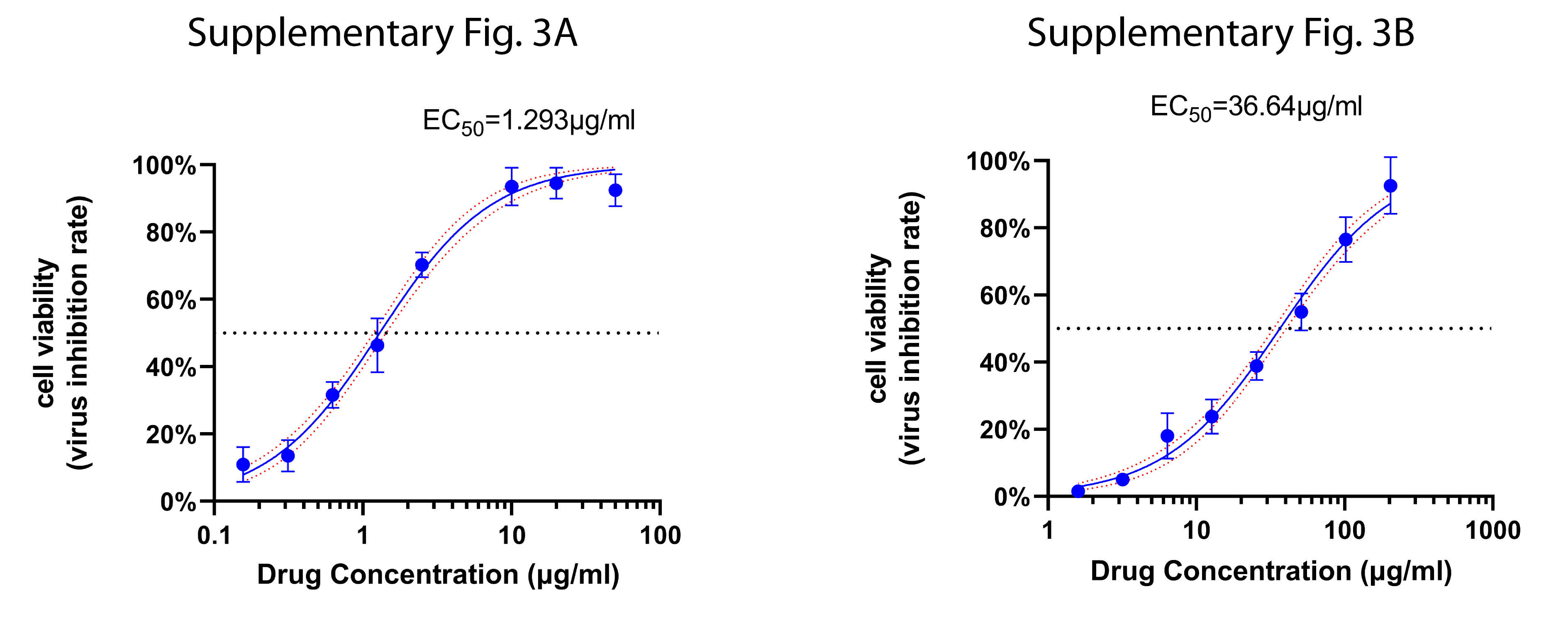

Supplement: Supplementary Figure 3 — Confluent MDCK cells cultured in 96-well plates were absorbed with 100TCID50 virus for 2 h, followed by co-culturing with baicalin (2-fold dilution from 204 μg/mL to 2.37 μg/mL) or oseltamivir (2-fold dilution from 20µg/mL to 0.156 µg/mL). The EC50 values were determined by the MTT method (Abcam) using GraphPad Prism 8. [file Image_3.tif]
